# Minnesota refugees diagnosed with tuberculosis disease, January 1993–August 2019

**DOI:** 10.1186/s12879-022-07327-0

**Published:** 2022-04-09

**Authors:** Kailey Urban, Blain Mamo, Dzung Thai, Alicia Earnest, Emily Jentes

**Affiliations:** 1 Infectious Disease Epidemiology, Prevention, and Control Division, Minnesota Department of Health, PO Box 64975, St. Paul, MN 55164 USA; 2 Division of Global Migration and Quarantine, U.S. Centers for Disease Control and Prevention, Atlanta, GA USA

**Keywords:** Tuberculosis, Screening, Refugee health, Surveillance

## Abstract

**Background:**

Refugees are screened for TB overseas using Technical Instructions (TIs) issued by the U.S. Centers for Disease Control and Prevention and after arrival during their refugee health assessment (RHA). We examined RHA results and TB outcomes of refugees to Minnesota.

**Methods:**

Demographic and RHA results for 70,290 refugee arrivals to Minnesota from January 1993 to August 2019 were matched to 3595 non-U.S. born individuals diagnosed with TB disease during that time.

**Results:**

Seven hundred fifty-nine (1.1%) were diagnosed with TB disease. Fifty-four percent were diagnosed within 2 years of U.S. arrival. Refugees screened using TIs implemented in 1991 were twice as likely to be diagnosed with TB disease within 1 year of arrival, compared to those evaluated using improved TIs implemented in 2007.

**Conclusion:**

Few refugees were diagnosed with TB disease during the period examined. Enhancements to overseas protocols significantly reduced the proportion of refugees diagnosed within 1 year of arrival.

## Background

In 2018, almost 70% of the 9029 new cases of tuberculosis in the United States were diagnosed in persons born outside of the United States [1]. In that year, the rate of tuberculosis disease was more than 14 times higher among non-U.S. born individuals compared to those born in the U.S. Also, 46% of tuberculosis cases in non-U.S.-born persons received a tuberculosis diagnosis ≥ 10 years after first arriving in the United States, consistent with a published estimate that reactivation of latent tuberculosis infection (LTBI) acquired overseas has been responsible for > 80% of domestic tuberculosis disease cases [2].

The Immigration and Nationality Act (INA) requires that all immigrants and refugees coming to the United States have an overseas medical examination; one aspect of that examination is to screen for infectious tuberculosis [3]. Overseas screening physicians are appointed by the Department of State (DOS) and are known as panel physicians; they perform the examination according to Technical Instructions (TIs), which are issued by the U.S. Centers for Disease Control and Prevention (CDC).

During January 1993–August 2019, two screening algorithms were followed overseas [4]. Beginning in 1991, applicants ≥ 15 years of age received a chest x-ray. If the chest x-ray findings were suggestive of pulmonary tuberculosis disease, three sputum specimens were required for acid-fast bacteria smear. Beginning in 2007, CDC started implementing a new TI that also requires children 2–14 years of age screened in countries where the World Health Organization (WHO)-estimated tuberculosis incidence rate is ≥ 20 per 100,000 to have a tuberculin skin test (TST) or interferon gamma release assay (IGRA) [5]. Children with a positive TST or IGRA are required to receive a chest x-ray. Applicants of any age with a chest x-ray suggestive of tuberculosis, or signs and symptoms of tuberculosis disease, or known HIV infection, must provide three sputum specimens for both smear and culture tests. The 2007 TIs require that cases diagnosed overseas complete therapy according to treatment guidelines established by the American Thoracic Society, CDC, and Infectious Diseases Society of America, with each dose delivered as directly observed therapy [6].

After U.S. arrival, individuals designated as refugees are eligible for benefits, including a recommended medical screening examination that addresses health conditions diagnosed overseas, identifies additional health concerns, and connects the individual with the U.S. healthcare system. The guidelines for this post-arrival refugee health assessment (RHA) are also established by CDC [7]. The overseas examination is meant to rule out infectious, pulmonary tuberculosis disease at the time of the examination. The domestic evaluation for TB as part of the RHA evaluates new refugee arrivals for pulmonary tuberculosis (in the event disease developed after the overseas examination was completed, or was missed upon initial examination), extrapulmonary tuberculosis, and LTBI, and treats those conditions, if found.

The objective of our analysis was to describe the demographic, tuberculosis screening history, and clinical characteristics of refugees who initially resettled in Minnesota and were diagnosed with tuberculosis disease in Minnesota from January 1993 to August 2019.

## Methods

Demographic and RHA screening results for 70,290 primary refugee arrivals to Minnesota from January 1993 to August 2019 were matched to 3595 non-U.S. born individuals diagnosed with tuberculosis disease during the same time period. Primary refugees include individuals with the following humanitarian designations who resettled initially in Minnesota upon U.S.-arrival or certification: refugees, asylees (U.S.-granted and derivative), parolees, Amerasians, and certified victims of human trafficking [8].

Demographic information and post-arrival RHA screening results were obtained from the Minnesota Department of Health’s Refugee Health Program database. Tuberculosis cases, including clinical information, risk factors, and co-morbidities at the time of diagnosis were obtained from the Minnesota Department of Health’s Tuberculosis Program surveillance system. Overseas screening results, including the TI used for overseas assessment (1991 versus 2007 TI), were obtained from CDC’s Electronic Disease Notification System or scanned records [9].

Records were matched on Alien Number, a unique identification number assigned to individuals immigrating to the United States. When Alien Number was unavailable, records were matched on name, date of birth, and U.S. arrival date. Records that matched on name, date of birth, and/or U.S. arrival date without the Alien Number were visually inspected to ensure they were an actual match. After the records were matched, personal identifiers were removed, and de-identified data were used for analysis.

All analyses were performed using Stata 15 software (StataCorp. 2017. Stata Statistical Software: Release 15. College Station, TX: StataCorp LLC). Figures and tables were created using Microsoft Excel (2016). Adjusted relative risks and 95% confidence intervals were calculated comparing the likelihood of being diagnosed with tuberculosis disease within 1 year of U.S. arrival by the type of overseas TI used. Arrivals from September 1, 2018 to August 31, 2019 were excluded from this portion of analysis, as they had not been in the U.S. for 1 year.

This analysis was determined to be non-research by a Minnesota Department of Health Human Subjects Advisor; institutional review board review was not required. As the data used for this analysis are collected as part of routine public health surveillance, and program staff from the Refugee Health and Tuberculosis Surveillance Programs conducted the analysis, no special permissions were required to access the data.

## Results

Among 70,290 primary refugee arrivals to Minnesota from 1993 to August 2019, 759 (1.1%) were diagnosed with tuberculosis disease in Minnesota at some point during this time period. These represented 766 diagnoses, as seven (0.9%) of the 759 individuals diagnosed with tuberculosis disease were counted as cases in Minnesota twice during this time period, indicating relapse of tuberculosis disease or a reinfection resulting in tuberculosis disease.

### Demographic characteristics

The median age at U.S. arrival among those diagnosed with tuberculosis disease was 20.6 years (interquartile range (IQR) 16.4–34.7) (Table 1) and the median age at tuberculosis disease diagnosis was 24.0 years (IQR 18.9–41.2). Four-hundred eighty (63.2%) cases were among Somalis, 84 (11.1%) among Ethiopians, 72 (9.5%) among Hmong, and the remaining 123 (16.2%) were from 18 other countries.

**Table 1:** Demographic characteristics of primary refugee arrivals to Minnesota and primary refugees diagnosed with tuberculosis disease in Minnesota, 1993–August 2019

Characteristic	All primary refugee arrivals to Minnesota*	Primary refugees diagnosed with TB disease in Minnesota
	N (%)	N (%)
Total	70,290 (100%)	759 (1.1%)
Nationality		
Somalia	24,011 (34.2%)	480 (63.2%)
Burma	8893 (12.7%)	28 (3.7%)
Hmong	8457 (12.0%)	72 (9.5%)
Ethiopia	5728 (8.2%)	84 (11.1%)
Liberia	4002 (5.7%)	30 (4.0%)
Other	19,199 (27.3%)	65 (8.6%)
Median age at U.S. arrival (IQR)	19.8 (11.4–32.7)	20.6 (16.4–34.7)

*67,044 (95%) of these arrived as primary refugees. The remaining 5% arrived as asylees, parolees, Amerasians, or certified victims of human trafficking

### Overseas screening

Among the 69,231 primary refugee arrivals to Minnesota from January 1, 1993 through August 31, 2018, 306 (0.4%) were diagnosed with tuberculosis disease within 1 year of U.S. arrival (Table 2).

**Table 2:** Risk of being diagnosed with tuberculosis disease within 1 year of U.S. arrival by overseas technical instruction (TI) used, 1993–August 2018

TI used	Total primary arrivals to MN	Diagnosed with tuberculosis disease w/in 1 year of U.S. arrival (%)	Unadjusted RR (95% CI)	Adjusted RR* (95% CI)
1991	49,742	266 (0.5%)	2.7 (1.9–3.8)	2.0 (1.5–2.9)
2007	19,276	40 (0.2%)	Ref	Ref
Total	69,018	306 (0.4%)		

*Adjusted for age at U.S. arrival and country of origin

Among all arrivals during this time period, 49,742 (72%) were evaluated overseas using the 1991 TIs and 19,276 (28%) were evaluated using the 2007 TIs. One-hundred sixty-six (< 1%) were not evaluated overseas for tuberculosis, as their status was granted in the United States (i.e. U.S.-granted asylees and certified victims of human trafficking) and the overseas TI type used could not be ascertained for an additional 47 (< 1%) including 4 individuals diagnosed with tuberculosis disease in the United States.

Two-hundred sixty-six (0.5%) of those evaluated using the 1991 TIs were diagnosed with TB in Minnesota within 1 year of U.S. arrival, compared to 40 (0.2%) evaluated using the 2007 TIs. After controlling for age at U.S. arrival and country of origin, individuals evaluated overseas using the 1991 TIs were approximately twice as likely to have been diagnosed with tuberculosis disease within 1 year of U.S. arrival compared to those evaluated using the 2007 TIs.

### Time from U.S. arrival to diagnosis and RHA screening results

**Fig. 1:**
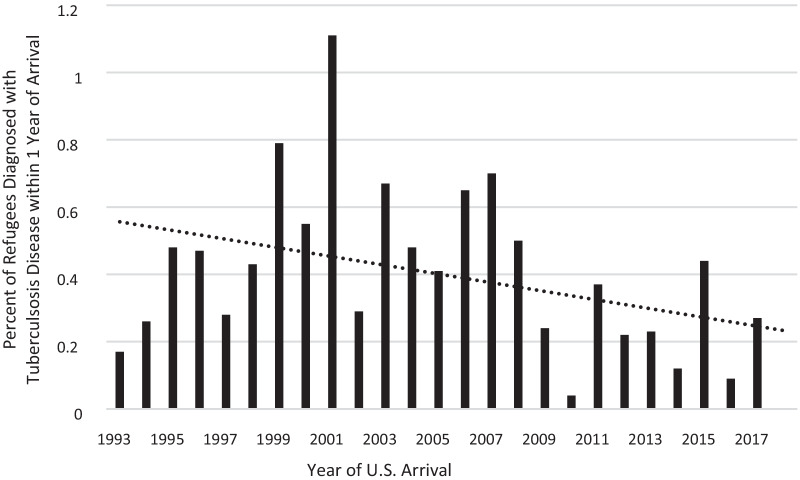
Percent of refugees diagnosed with tuberculosis disease within 1 year of U.S. arrival

The proportion of total refugee arrivals diagnosed with tuberculosis disease within 1 year of U.S. arrival generally decreased over time (Fig. 1). The highest proportion was in 2001, with 31 (1.1%) of 2763 refugees diagnosed with tuberculosis disease within 1 year. Among refugee arrivals from January to August 2018, none were diagnosed with tuberculosis disease within 1 year.

The majority (53.5%) of tuberculosis disease cases were diagnosed within 2 years of U.S. arrival (Table 3). The time from U.S. arrival to tuberculosis diagnosis ranged from less than 1 month to 24.8 years. The median time spent in the U.S. from arrival through August 31, 2019 for those diagnosed with tuberculosis disease was 14.6 years (IQR 8.9–19.6 years).

**Table 3:** Time from U.S. arrival to tuberculosis disease diagnosis

Time from U.S. arrival to tuberculosis diagnosis (years)	Number* (% of cases)	Cumulative %
< 1	306 (39.9)	39.9
1–1.9	104 (13.6)	53.5
2–4.9	136 (17.8)	71.3
5–9.9	122 (15.9)	87.2
10–14.9	66 (8.6)	95.8
15–19.9	21 (2.7)	98.5
20–24.9	11 (1.4)	100.0
Total	766 (100%)	

*Total tuberculosis disease diagnoses. Seven individuals were diagnosed with tuberculosis disease twice during this time period

The median time from U.S. arrival to first tuberculosis disease diagnosis was 1.61 years (IQR 0.43–5.51) (Table 4). Of the 759 primary refugees diagnosed with tuberculosis disease in Minnesota, 242 (31.8%) were first diagnosed during the post-arrival Refugee Health Assessment (RHA). Of the remainder, 270 (35.6%) were diagnosed with latent tuberculosis infection (LTBI) at their RHA, 103 (13.6%) were diagnosed with no infection or disease at the RHA, 7 (0.9%) had a history of tuberculosis treatment overseas and were re-diagnosed with tuberculosis disease sometime after the RHA, and 137 (18%) were not evaluated for tuberculosis during the RHA, or did not receive a RHA.

**Table 4:** Refugee health assessment TB screening results and time from U.S. arrival to first tuberculosis diagnosis

Refugee health assessment tuberculosis screening result	N (%)	Median time (years) from U.S. arrival to first tuberculosis disease diagnosis (IQR)
TB disease	242 (31.8%)	0.35 (0.23–0.59)
Latent TB infection*	270 (35.6%)	3.70 (1.65–7.01)
Treated for TB disease overseas	7 (0.9%)	1.76 (1.33–3.27)
No TB Infection or disease**	103 (13.6%)	5.51 (2.33–11.3)
Not screened for TB at RHA or did not receive RHA	137 (18.0%)	3.16 (0.87–7.29)
Total	759 (100%)	1.61 (0.43–5.51)

*A positive TST and/or IGRA and tuberculosis disease was ruled out

**A negative TST and/or IGRA

Among the 270 diagnosed with LTBI at the RHA who later went on to develop tuberculosis disease, 179 (66%) had initiated LTBI treatment and 82 (30%) had reported LTBI treatment completion. Sixty (22%) did not initiate LTBI treatment, and treatment initiation and completion statuses were unknown for an additional 31 (11%).

The median time from U.S. arrival to first diagnosis of tuberculosis disease differed by screening outcome during the RHA. Consistent with the timing of the RHA, the median time from U.S. arrival to first tuberculosis diagnosis was 0.35 years among those diagnosed at the RHA. The median time from U.S. arrival to tuberculosis disease among those diagnosed with LTBI at the RHA was 3.70 years, 1.76 years among those with a history of tuberculosis treatment overseas, 5.51 years among those diagnosed with no tuberculosis infection or disease, and 3.16 years among those not evaluated at the RHA or who did not receive a RHA.

Among the seven who were diagnosed with tuberculosis disease twice during this time period, the median time from first diagnosis to second diagnosis was 3.36 years (range 2.24–15.58 years).

### Clinical characteristics and risk factors

Among the 766 cases of tuberculosis disease diagnosed among primary refugees during this time period, 398 (52%) had pulmonary disease, 305 (40%) had extrapulmonary, and 63 (8%) had both pulmonary and extrapulmonary. Nineteen cases (2%) were resistant to at least isoniazid, 18 (2%) were resistant to at least rifampin, and 11 (1%) were resistant to at least isoniazid and rifampin and thus had MDR (multidrug-resistant) tuberculosis. One individual diagnosed with tuberculosis disease twice during this time had MDR tuberculosis both times.

Seventy-three (9.5%) had at least one immunosuppressive risk factor at the time of TB diagnosis (Table 5). Thirty-six (4.7%) had diabetes at the time of diagnosis, 16 (2.1%) were HIV positive, 4 (0.5%) were underweight or malnourished (not related to TB), 14 (1.8%) were on immunosuppressive therapy or had another immunosuppressive condition, 8 (1%) were experiencing end-stage renal disease. Additionally, 10 (1.3%) had a history of alcohol or drug abuse in the previous year, and 8 (1%) had experienced homelessness in the previous year.

**Table 5:** Risk factors and co-morbidities at the time of diagnosis in refugee arrivals diagnosed with tuberculosis disease

Risk factor/co-morbidity*	Number****	%
Any immunosuppressive risk factor**	73	9.5
Diabetes	36	4.7
HIV	16	2.1
Underweight/Undernutrition (not due to TB disease)	4	0.5
Immunosuppressive therapy or other immunosuppressive condition***	14	1.8
End-stage renal disease	8	1.0
Alcohol or drug abuse in past year	10	1.3
Homelessness in past year	8	1.0
Total	766	100

*Patients could have ≥ 1 risk factor or co-morbidity

**Conditions or therapies that increase risk for progression from LTBI to tuberculosis disease

***Includes TNFα antagonist therapy and post-organ transplantation

****Total tuberculosis disease diagnoses. Seven individuals were diagnosed with tuberculosis disease twice during this time period, but could have experienced different risk factors and co-morbidities at each diagnosis

## Discussion

Only 759 (1.1%) of the 70,290 primary refugee arrivals to Minnesota during this time period were ever diagnosed with tuberculosis disease in Minnesota. This is similar to the proportion found in another study looking at U.S.-bound immigrants and refugees from 1999 to 2005 [10]. The majority were diagnosed within 2 years of U.S. arrival, consistent with findings from another study, which found TB case rates to be the highest among non-U.S. born persons who had entered the United States most recently [11].

In this analysis, compared to refugees evaluated using 2007 TIs, refugees evaluated using the 1991 overseas TIs had approximately twice the likelihood of being diagnosed with tuberculosis disease within 1 year of U.S. arrival, after adjusting for country of origin and age at U.S. arrival. This is consistent with other studies, which have found a reduction in the incidence of tuberculosis among newly arrived, non-U.S. born persons following the implementation of the 2007 TI, which introduced culture-based screening [10, 12–16]. As the majority of cases of tuberculosis disease among refugees included in this analysis were diagnosed within 2 years of U.S. arrival, the updated TIs have the potential to significantly reduce the incidence of tuberculosis diagnosed among refugees and immigrants going forward.

In addition to the robust overseas screening protocols, the post-arrival RHA provides another opportunity to identify and treat refugees with tuberculosis disease that developed subsequently, and to prevent tuberculosis disease by screening and treating new arrivals for LTBI. Among the refugees diagnosed with tuberculosis disease in Minnesota, 32% were diagnosed at their post-arrival RHA. Thirty-six percent were diagnosed with LTBI at the post-arrival RHA, the majority of whom did not initiate and/or complete treatment. Treatment of LTBI is an important step in the prevention of tuberculosis disease, as the majority of TB cases in the United States are attributable to reactivation of LTBI [2]. Among all primary refugee arrivals to Minnesota from 2010 to 2017, 89% of those diagnosed with LTBI at the post-arrival RHA successfully completed LTBI treatment [12]. The LTBI completion rates in studies among refugees resettling in the United States, Canada, or Australia varies widely, but are generally lower than 89% [17–23]. One study found that refugees with LTBI are more likely to complete LTBI treatment compared to other non U.S.-born and U.S.-born populations [24]. Minnesota maintains a strong program for screening newly arrived refugees and promoting treatment of LTBI through coordinated efforts with screening clinics and local public health [25].

Compared to all Minnesota tuberculosis cases, refugees in this population had fewer co-morbidities at the time of diagnosis [26]. One study found that non U.S.-born individuals diagnosed with tuberculosis disease ≥ 10 years after U.S. arrival were more likely to have immunocompromising conditions compared to those diagnosed < 10 years after U.S. arrival [27]. As this population ages and develops immunocompromising conditions such as diabetes, the likelihood of the reactivation of LTBI may increase [28], underscoring the need for prevention through identification and treatment of LTBI. To minimize the risk of progression to tuberculosis disease, it will be important to evaluate and treat all at risk in this population as they age and develop risk-factors associated with the development of tuberculosis disease.

This evaluation had several limitations. First, because we only utilized data from Minnesota, it is possible that refugees who arrived during this time period moved out of Minnesota and developed tuberculosis disease in another state or country and would not have been included as a case in this analysis. Additionally, for refugees who developed tuberculosis disease after their post-arrival RHA, it is unknown whether their exposure occurred overseas or in the United States. And for those who were treated for LTBI at the post-arrival RHA and subsequently developed tuberculosis disease, it is unknown whether treatment outcome was inaccurately reported, the treatment did not successfully prevent progression to tuberculosis disease, or a subsequent exposure occurred after treatment. Also, this analysis did not control for the decrease of incidence of TB in refugees’ countries of origin. Finally, some populations that are being resettled to the United States for the first time, such as arrivals from Bhutan, Burma, and the Democratic Republic of the Congo, have not been in the United States long enough to be followed for the development of tuberculosis over time, as compared to refugee populations that arrived earlier during this time period, such as arrivals from Somalia, Liberia, and Laos.

Similar evaluations have only been able to follow refugees up to U.S. arrival or within 1 year of arrival [12, 14, 15]. The Minnesota Department of Health’s Refugee Health Program and Tuberculosis Program routinely share data to identify refugees with suspected or confirmed tuberculosis disease and establish screening histories. The strong partnership between the two programs allowed us to match the refugee health and tuberculosis surveillance data, and identify diagnoses of tuberculosis disease that occurred years after U.S. arrival.

## Data Availability

The datasets analyzed during the current study are not publicly available as they contain identifiable data about vulnerable populations. However, de-identified data pertaining to this study are available from the corresponding author on reasonable request.
